# Closure of Mesenteric Defects during Roux-en-Y Gastric Bypass Fails to Reduce Internal Herniation

**DOI:** 10.1007/s11695-025-07969-4

**Published:** 2025-07-10

**Authors:** Annick E. Taselaar, Ron W. F. de Bruin, T. Martijn Kuijper, Erwin van der Harst, René A. Klaassen

**Affiliations:** 1 Maasstad Ziekenhuis, Rotterdam, Netherlands; 2 Erasmus MC, Rotterdam, Netherlands; 3 Erasmus MC, Rotterdam, Netherlands

**Keywords:** Internal herniation (IH), Mesenteric defect (MD), Jejunojejunostomy (JJ) kinking, Roux-en-Y gastric bypass (RYGB), Bariatric surgery, Endohernia stapling, Reoperation

## Abstract

**Introduction:**

The rising obesity rates have led to an increased demand for bariatric surgery, particularly laparoscopic Roux-en-Y gastric bypass (LRYGB). While effective, LRYGB carries risks such as internal herniation (IH). This study evaluates the impact of routinely closing mesenteric defects (MD) during LRYGB on IH incidence and postoperative outcomes in a high-volume bariatric center.

**Methods:**

We conducted a retrospective cohort study of 6896 and 1903 LRYGB procedures before and after implementing routine MD closure respectively. We analyzed incidence of IH, kinking at the jejunojejunostomy (JJ), ICU admissions, hospital stay, postoperative pain relief, and the diagnostic value of CT scans.

**Results:**

The incidence of IH in the closure group (2.84%) was not significantly different from the non-closure group (2.80%). Postoperative pain relief rates were similar between the groups. However, routine MD closure led to the occurrence of JJ kinking (0.84%), which was not present in the non-closure group, resulting in prolonged hospital stays and ICU admissions. CT scans were predictive for IH but had limitations.

**Conclusion:**

Routine closure of MD during LRYGB did not reduce IH incidence but introduced the new complication of JJ kinking. Postoperative pain relief rates were unaffected by MD closure. Our findings highlight the need for further research on alternative closure methods and improved diagnostic strategies to optimize surgical outcomes.

**Graphical Abstract:**

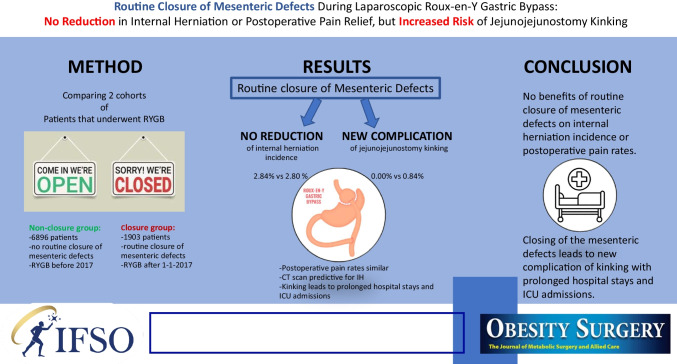

## Introduction

With the rise in obesity rates, the demand for bariatric surgery has increased dramatically, a trend expected to continue as the obesity epidemic persists. Bariatric surgery, particularly laparoscopic Roux-en-Y gastric bypass (LRYGB), has proven effective not only in promoting significant weight loss but also in improving obesity-related comorbidities such as type 2 diabetes, hypertension, and sleep apnea. LRYGB has become the standard of care in bariatric surgery and is performed over 200,000 times per year worldwide [1]. This procedure combines restrictive and malabsorptive components, creating a small gastric pouch (~ 25 cc) that connects to the jejunum and bypasses the upper small intestine to induce malabsorption.

Despite its benefits, LRYGB is not without complications, with internal herniation (IH) being one of the most serious and challenging to manage. IH occurs when the intestines herniate through mesenteric defects (MD) created as a result of the anatomical changes by the surgery. IH can lead to small bowel obstruction and is considered the most common cause of abdominal pain after LRYGB. Literature reports varying rates of IH up to 15% on the long-term after RYGB [2–5]. The presence of intermittent IH, which may not always be visible on imaging, poses diagnostic challenges and contributes to unexplained abdominal pain. This uncertainty can necessitate exploratory relaparoscopy, where the MD can be closed to prevent recurrent IH, resulting in postoperative pain relief.

To address the risk of IH, Gislason et al. introduced a new technique in 2012 using endohernia staplers to close the MD during primary surgery, aiming to reduce the incidence of postoperative IH [6]. Inspired by this approach, our team visited Gislason’s center in 2016, where our surgeons received hands-on training in the technique. Following this visit and his pivotal study, we decided to routinely close the MD in all LRYGB procedures performed at our institution starting January 1, 2017. Prior to this change, the incidence of reoperations for suspected IH was 2.8% when the MD were routinely left open, with mixed outcomes in terms of pain relief [7]. Although literature suggests that closing the MD could be beneficial, there is also a risk that these defects may reopen after substantial weight loss due to the loss of intra-abdominal fat [8].

While MD closure has been associated with a potential reduction in IH, it is not without drawbacks. Closing the MD introduces the risk of early postoperative complications, such as obstruction at the jejunojejunostomy (JJ) site, known as kinking. This can lead to severe complications, including blowout of the biliopancreatic limb, significant morbidity, ICU admission, and even mortality [9, 10]. Therefore, it is crucial to evaluate the effectiveness of routinely closing the MD by weighing the reduced risk of IH against the potential complications associated with kinking. This study aims to assess the comprehensive impact of routinely closing the MD in our high-volume bariatric surgery center, focusing on both the reduction of IH and the potential risks of early postoperative obstruction.

## Methods

### Study Design

This study is a retrospective cohort study. We evaluated the effect of routinely closing the MD, a surgical technique adjustment implemented at our institution on January 1, 2017, by reviewing patient files from surgeries before and after the implementation of this technique. To identify reoperations within the follow-up period, the hospital’s Business Intelligence (BI) team provided a comprehensive list of all patients who underwent primary LRYGB during the study period and were subsequently reoperated. Subsequently, we screened this list to identify patients who had undergone diagnostic reoperations (laparoscopy or laparotomy) for suspected IH or JJ kinking. We defined MD (mesenteric defect) as defects in the mesentery associated with IH. Specifically, we included both the JJ (jejunojejunostomy) defect and Petersen’s defect:

- **The JJ defect** occurs at the site of the anastomosis between the two parts of the jejunum, where the mesentery is divided during the creation of the JJ anastomosis. This division creates an orifice that can allow the bowel to herniate.
- **Petersen’s defect** refers to the orifice between the mesocolon and the mesentery of the proximal jejunum, caused by the ante-colic route used for the gastrojejunostomy (GJ) anastomosis.
- **Kinking** was defined as iatrogenic obstruction of the JJ anastomosis resulting from the specific technique used to close the JJ defect.

The first author conducted a detailed review of the medical files of these patients, extracting relevant data and ensuring adherence to inclusion criteria. To enhance validity and transparency, author RK independently cross-checked the reviewed files. Although the analysis was conducted retrospectively, all patient files were prospectively updated during follow-up, with data entered directly over the years. Our own team performed follow-up, ensuring continuity and accuracy in patient care. Additionally, all patients were systematically included in the Dutch Audit for Treatment of Obesity (DATO), guaranteeing comprehensive data collection and monitoring during the 5-year postoperative follow-up [11].

### Definition of IH and diagnostic approach

In this study, we used the following definitions for IH:

- **Suspected IH**: Patients who undergo reoperation for suspected IH based on clinical symptoms, imaging findings, or both.
- **Confirmed IH:** Patients with visible herniation observed and confirmed during reoperation. This includes any herniation observed perioperatively.
- **Intermittent IH:** Patients who have clinical symptoms consistent with IH suspicion, with or without signs on CT, but no internal herniation observed during reoperation. These patients report complete pain relief 3 months after surgery, suggesting that the closure of the mesenteric defect (MD) during reoperation addresses symptoms consistent with intermittent IH.

### Patient Population

The Maasstad Hospital in Rotterdam is a high-volume bariatric surgery center in the Netherlands, performing approximately 600 bariatric procedures annually, with over 80% being LRYGB. This study included all primary LRYGBs performed between January 1, 2017, and September 30, 2021, following the routine closure of MD. We reviewed follow-up data until April 1, 2024. All patients met the standard inclusion criteria for bariatric surgery as defined by the International Federation for the Surgery of Obesity and Metabolic Disorders (IFSO) [12]. Patients who underwent reoperation during the follow-up period for suspected IH or suspected kinking of the JJ were selected for a detailed patient file review. Exclusions were made for patients in whom other conditions, such as obstructing adhesions, were diagnosed during reoperation.

### Groups

To compare outcomes with those before the routine closure of the MD, we included patients from our previous study on IH as the non-closure group [7]. We divided the current cohort into two subgroups within the closure group:

- **Non-closure group:** Patients who underwent primary LRYGB without routine closure of the mesenteric defect (MD), as described in our previous study [7].
- **Closure group:** Patients who underwent primary LRYGB with routine closure of the MD, divided into the following subgroups:
  o **Closure-IH group:** Patients who underwent reoperation for suspected IH, based on clinical symptoms and/or imaging findings
  o **Closure-kinking group:** Patients who underwent reoperation for suspected kinking of the jejunojejunostomy (JJ), based on clinical symptoms and/or imaging findings.

### Radiology

We assessed the *sensitivity* and *specificity* of CT scans in our cohort to evaluate their diagnostic performance in detecting IH, but did not use CT results to classify patients. A radiologist and an experienced bariatric surgeon evaluated the CT scans. We considered a CT scan positive for IH if we observed a swirl sign of mesenteric vessels with an estimated rotation of at least 180°. We also included CT scans exhibiting other signs of IH, even in the absence of a swirl sign, in the analysis. These other signs included small bowel obstruction, clustered loops, mushroom sign, hurricane eye sign, small bowel behind the superior mesenteric artery, right-sided anastomosis, enlarged nodes, venous congestion, and mesenteric edema, as described by Ederveen et al. [13]. We considered CT scans positive for kinking if we observed dilatation of the small bowel in the alimentary limb or bilio-pancreatic limb due to kinking of the JJ, or if signs of obstruction at the level of the JJ anastomosis without other explanatory anomalies were present.

### Surgical Procedure

A gastric pouch of approximately 25 cc was created using an endo-GIA, starting 4 cm from the esophagogastric junction and advancing toward the angle of His. Next, we dissected the omentum with the Harmonic Ace (ETHICON, USA). Subsequently, the transverse colon was elevated to identify the ligament of Treitz. We measured 50 cm of jejunum, positioned it in a supracolic and supragastric orientation, and created a GJ. We closed the dorsal aspect of the anastomosis with an endo-GIA and the ventral side with a continuous V-lock suture. To assess the integrity of the anastomosis for leaks, a methylene blue test was performed.

We then measured 150 cm of jejunum to establish the JJ which was closed with an endo-GIA on the dorsal side and a continuous V-lock suture on the ventral side. Finally, we transected the jejunum between the two anastomoses to create a Roux-en-Y configuration.

### Method of Closing

After we completed the final transection to create the Roux-en-Y configuration, we brought the JJ to the right side to reveal the mesenteric defect. We closed the defect using 8–10 endohernia staples (ETHICON, USA), starting at the cranial side of the JJ and progressing toward the caudal part of the defect.

Next, we elevated the transverse colon to expose the subcolic space behind the alimentary limb, known as Petersen’s space. We closed Petersen’s space by stapling the mesentery of the transverse colon to the mesentery of the alimentary limb.

### Statistical Analysis

We performed statistical analysis using the Stata software package (Stata, version 15.1, StataCorp). We used univariate logistic regression to investigate predictors of IH during reoperation and postoperative pain relief. We used multivariable logistic regression with backward elimination (*p* < 0.05) to identify independent predictors of IH during reoperation.

We summarized baseline characteristics, surgical aspects, and the incidence of IH and kinking using descriptive statistics. To compare groups, we applied independent *t*-tests for normally distributed data, Mann–Whitney *U* tests for non-normally distributed data, and chi-square tests for categorical data. We considered a *p*-value of < 0.05 to be statistically significant.

## Results

### Routine MD Closure Does Not Reduce IH Incidence

We studied a previous cohort of 6896 patients who underwent RYGB without closure of MD (non-closure group). Of these, 193 patients (2.80%) underwent reoperation for suspected IH [7]. We included 1903 patients in the current retrospective cohort who underwent RYGB between January 1, 2017, and September 30, 2021, with routine closure of MD. We conducted follow-up on these patients until April 1, 2023, with a median follow-up period of 51.7 [40.5–63.1] months. Among these patients, 54 required reoperation for suspected IH (closure-IH group) and 16 cases for suspected kinking of the JJ (closure-kinking group). The rate of relaparoscopy for suspected IH in the closure group was 2.84% (54 out of 1903), which was not significantly different compared to the incidence before routine closure of the MD (2.84% vs. 2.80%, *p* = 0.928).

### No Significant Differences in Patient Characteristics Between Closure and Non-closure Groups

Table 1 summarizes the characteristics of patients who underwent reoperation, highlighting comparisons between the following groups:

- **Non-closure**: Patients who underwent RYGB without mesenteric defect closure and subsequently required reoperation for suspected IH.
- **Closure-IH**: Patients who underwent RYGB with mesenteric defect closure and subsequently required reoperation for suspected IH.
- **Closure-kinking**: Patients who underwent RYGB with mesenteric defect closure and subsequently required reoperation for suspected JJ kinking.

**Table 1:** Patient characteristics of reoperation groups

Demographics	Non-closure (*n*= 193)	Closure-IH (*n* = 54)	Closure-kinking (*n* = 16)	*Non-closure vs Closure-IH p-value*	*Closure-IH vs Closure-kinking p-value*
Age at reoperation	41.5 (± 9.6)	44.6 (± 10.4)	45.8 (± 12.5)	0.041	0.695
Gender female	171 (88.6%)	42 (77.8%)	16 (100%)	0.041	0.038
BMI before RYGB	42.4 (± 5.4)	40.9 (± 4.8)	38,3 (± 4.7)	0.067	0.058
BMI at reoperation	28.9 (± 5.7)	27.3 (± 4.9)	37.6 (± 5.3)	0.071	< 0.001
BMI loss between RYGB and reoperation	13.6(± 5.3)	13.6(± 4.4)	0.18 [0.0–1.0]	0.949	< 0.001
TWL between RYGB and reoperation	38.7 (± 16.4)	39.0 (± 13.3)	0 [0–3.25]	0.550	
% TWL	31.4 (± 11.4)	31.9(± 9.5)	2.1 (± 4.0)	0.739	< 0.001
Interval RYGB-reoperation (months)	18.2 [11.4–30.7]	16.6 [9.4–31.9]	0.41 [0.33–0.54]	0.592	< 0.001
Acute presentation	72 (37.3%)	40 (74.1%)	16 (100%)	< 0.001	0.023
Smoking	52 (26.9%)	10 (18.5%)	0 (0%)	0.207	0.063

#### Closure-IH Group

Patients in the closure-IH group had a mean age of 44.6 ± 10.4 years, with 77.8% being female. Their mean BMI before RYGB was 40.9 ± 4.8. The median interval between RYGB and reoperation was 16.6 [9.4–31.9] months. The mean total weight loss (TWL) in the closure-IH group before relaparoscopy was 39 (± 13.3) kg, which was not significantly different from the TWL before relaparoscopy in the non-closure group (38.7 ± 16.4, *p* = 0.550) (Table 1).

Before reoperation, 18.5% of patients reported smoking. 74.1% of reoperations were performed in an acute setting. Surgical aspects of reoperation are summarized in Table 2. Surgery confirmed IH in 23 of 54 patients, representing 1.21% of the total RYGB cohort (*n* = 1903), which was comparable to the percentage in patients who did not undergo routine closure (1.21% vs. 1.71%, *p* = 0.122). We observed herniations through the defect of the JJ more frequently than through Petersen’s space (57% and 39% of all observed herniations, respectively) but this was not significantly different (*p* = 0.405). Additionally, 10 patients (18.5%) who underwent reoperation for suspected IH required a subsequent reoperation due to either persistent symptoms or recurrence of symptoms.

**Table 2:** Surgical aspects of reoperation

Closure-IH group		
Surgery aspects	Number (*n* = 54)	%
Conversion to laparotomy	1	1.9%
Internal herniation present in reoperation	23	42.6%
Petersen’s hernia	9	16.7%
JJ-stomy hernia	13	24.1%
Petersen’s and JJ-stomy	1	1.9%
Closure technique RYGB		
Staples	52	96.3%
Non absorbable Suture	0	0%
Absorbable suture	0	0%
Unknown	2	3.7%
Closure technique reoperation		
Non absorbable suture	45	83.3%
Absorbable suture	2	3.7%
Staples	1	1.9%
Unknown	1	1.9%
Postoperative pain relief	38	70.4%
Additional reoperation for suspected IH	10	18.5%

#### Closure-Kinking Group

Patients in the closure-kinking group had a mean age of 45.8 ± 12.5 years; all were female. Their mean BMI before RYGB was 38.3 ± 4.7. The median interval between RYGB and reoperation was 0.41 months (12.5 days). All reoperations in this group were performed in an acute setting. No patients reported smoking before reoperation.

### High Rate of Reopened MDs After Routine Closure

During RYGB, we closed MDs using endohernia stapling (ETHICON, USA) in 52 cases (96.3%). The closure technique was unknown in 2 patients. Upon reoperation, one or both MD were found to be reopened in 49 out of 54 patients (90.7%). Among the 31 patients (83.9%) who did not present with perioperative IH, 26 had open mesenteric defect(s). We observed no correlation between TWL and the status (open or closed) of MD.

### No Difference in Postoperative Pain Relief Between Closure and Non-closure Groups

Among the 54 patients who underwent relaparoscopy for suspected IH in the closure group, 38 (70.4%) reported complete pain relief postoperatively, a rate not significantly different from the 77.2% (146 out of 189 patients) in the cohort before routine closure of MD (*p* = 0.299). Of the 49 patients with open MD, 34 (69.4%) reported complete pain relief postoperatively. Among those with IH perioperatively, 65.2% of patients with open MD reported postoperative pain relief compared to 73.1% when no IH was present perioperatively (*p* = 0.551). Four out of 5 patients (80%) with closed MD reported complete pain relief postoperatively.

### CT Scan Show Variable Sensitivity for IH Detection

CT scans demonstrated varying sensitivity and specificity for detecting internal hernias (IH). The swirl sign showed 30.4% sensitivity with high specificity (93.3%), while overall sensitivity increased to 87.0% when any IH signs were present, though specificity remained lower at 66.7%. These results indicate that CT scans, while useful, may not always detect IH, particularly in cases without clear signs such as the swirl sign.

Table 3 summarizes the sensitivity and specificity values for different CT findings related to IH detection. Despite negative CT results in some patients, clinical presentation suggesting intermittent IH prompted the decision to proceed with relaparoscopy. Among the 23 patients with negative CT scans, only 3 (13%) had IH perioperatively, demonstrating that clinical judgment remains crucial in managing suspected IH cases. Interestingly, 74% of these patients reported complete pain relief postoperatively, suggesting that, despite negative imaging, the surgical intervention was beneficial. In contrast, when CT scans showed any signs of IH (*n* = 30), 66.7% of patients reported complete pain relief. Furthermore, among the 9 patients with a positive swirl sign on CT, 56% reported complete pain relief, highlighting the potential for CT signs to aid in predicting postoperative outcomes.

**Table 3:** CT scan findings and clinical significance in detecting IH

CT scan finding	Sensitivity (%)	Specificity (%)
Swirl sign	30.4%	93.3%
Other signs of IH	81.3%	71.4%
Any signs of IH (overall)	87.0%	66.7%
Negative CT scan (IH present)	13%	-

### More Acute Reoperations in the Closure Group

The closure-IH cohort had 74.1% of reoperations performed in an acute setting, while the non-closure cohort had only 37.3% of reoperations in an acute setting (*p* < 0.001). All reoperations for suspected kinking took place in an acute setting.

### CT Scan Findings and Acute Surgery Setting Are Independent Predictors of IH

In the univariate analysis, a swirl sign on preoperative CT significantly predicted perioperative IH (OR 26.7 [2.2–326.5]), along with other signs of IH on CT (OR 10.9 [2.4–48.5]). Additionally, reoperations performed in an acute setting identified IH more frequently perioperatively. The results of the univariate analysis are shown in Table 4.

**Table 4:** Predictors for IH perioperatively

Univariate analysis			
Variable	OR	95% CI	*p *value
Age > 45	0.98	[0.33–2.89]	0.967
Female gender	1.05	[0.29–3.85]	0.941
BMI ≥ 30	1.82	[0.52–6.41]	0.350
BMI loss ≥ 12	0.63	[0.20–1.99]	0.438
% TWL > 40	0.27	[0.05–1.44]	0.126
Acute surgery	15.89	[1.89–133.33]	0.011
CT any signs of IH	13.33	[3.19–55.79]	< 0.001
CT swirl	6.13	[1.13–33.10]	0.035

No significant difference in the incidence of IH occurred between men and women perioperatively. Furthermore, a BMI < 30, TWL > 40 kg, or a BMI reduction > 12 points did not predict perioperative IH.

In the multivariable analysis, the swirl sign on preoperative CT and other signs of IH on CT remained significant predictors of perioperative IH. The association with an acute surgery setting showed borderline significance (*p* = 0.08) (Table 5).

**Table 5:** Predictors for IH perioperatively

Multivariable analysis			
Variable	OR	95% CI	*p* value
Acute surgery	7.57	[0.77–74.28]	0.082
CT signs of IH other than swirl	8.42	[1.76–40.22]	0.008
CT swirl	15.48	[1.22–196.77]	0.035

### No Predictive Factors for Pain Relief 3 months Postoperatively

IH presence during reoperation did not affect pain relief at 3 months postoperatively. Similarly, whether MD were reopened or remained closed did not significantly predict pain relief at 3 months postoperatively. Additionally, variables including age > 45 years, sex, weight loss (BMI reduction > 12 points, TWL > 40 kg, BMI prior to reoperation < 30), and surgical setting (acute or elective) did not influence postoperative pain relief. Moreover, a positive preoperative CT for IH (swirl sign or other signs of IH) did not impact postoperative pain relief. The results of the univariate analysis are shown in Table 6.

**Table 6:** Predictors for pain relief 3 months postoperatively

Univariate analysis			
Variable	OR	95% CI	*p* value
Age > 45	1.29	[0.40–4.16]	0.675
Female gender	0.74	[0.17–3.21]	0.691
BMI ≥ 30	1.55	[0.36–6.59]	0.554
BMI loss ≥ 12	1.30	[0.38–4.41]	0.674
% TWL > 40	4.66	[0.54–40.28]	0.162
Acute surgery	0.57	[0.14–2.39]	0.438
CT any signs of IH	0.71	[0.21–2.35]	0.570
CT swirl	0.47	[0.11–2.04]	0.313
IH perioperatively	0.65	[0.20–2.11]	0.476
Open MD	0.57	[0.06–5.51]	0.624

### Kinking of the JJ Leads to Prolonged Hospitalization and More ICU Admissions

Sixteen patients (0.84% of the total RYGB cohort) underwent relaparoscopy for suspected kinking of the JJ, while no relaparoscopies for this condition were performed in the non-closure group (*p* < 0.001). A preoperative CT scan was performed in all 16 cases, yielding a sensitivity of 73% and a specificity of 0%, as there were no true negative scans. Three scans were false negatives. Kinking of the JJ was confirmed in 11 patients, representing 0.6% of the total RYGB cohort. The TWL in this group was not recorded, as the reoperation occurred before their first scheduled postoperative outpatient follow-up visit, and thus no new weight was documented between the primary surgery and the reoperation.

#### Hospitalization

The median length of stay for all patients hospitalized due to suspected IH or suspected kinking was 2 days, with a range from 1 to 42 days. Specifically, patients with suspected IH had a median stay of 1 day, while those with suspected kinking stayed for a median of 8 days.

#### ICU Admission

Three patients required ICU admission during hospitalization for suspected kinking, with stays lasting 4, 10, and 1 day, respectively. In contrast, 1 patient in the IH group was admitted to the ICU for 1 day. The total number of ICU admission days was significantly higher in the kinking group compared to the IH group (*p* = 0.03). These findings suggest that kinking may result in greater morbidity than IH.

## Discussion

*Contrary to our expectations*, our study demonstrates that routine MD closure during LRYGB does not reduce the risk of clinically significant IH as there was no reduction in reoperations for suspected IH, confirmed IH during those reoperations, or intermittent IH based on pain relief rates. Closure, however, introduces a new complication, so called *kinking* of the jejunojejunostomy. This occurred in 0.84% of patients, resulting in high morbidity, including prolonged hospitalization and increased ICU admissions. Ten patients (18.5%) in the closure group who underwent reoperation required a second reoperation for suspected IH. This suggests that, in some cases, symptoms may recur or persist despite initial surgery. Finally, our study revealed that CT scans are insufficient as sole diagnostic tool to decide whether IH has occurred, likely due to the intermittent nature of this phenomenon, which contributes to both the variable sensitivity and lower specificity of the scans. This underscores the complexity of IH diagnosis and management.

Contrary to recent findings, including a 2020 meta-analysis advocating routine closure, we found no significant reduction in IH incidence following closure [14]. However, only three randomized controlled trials have investigated the effect of closing MD, yielding conflicting results. Sternberg et al. conducted a large study in over 2500 patients with a 10-year follow-up, and reported a reduction in IH incidence from 12.9 to 3.8% > 30 days postoperatively, along with an increase in JJ kinking to 1.3%, similar to our findings [15]. Kristensen et al. reported a reduced risk of IH after closure with clips in a smaller study of 401 patients, with high IH rates both with and without closure up to 15.5% and 6.5% respectively. Kinking was not mentioned, but 1.5% of the closure group underwent reoperation for JJ torsion within 30 days [16]. Rosas et al. found no difference in IH incidence between closure and non-closure groups after a 3-year follow-up in a study of 105 patients. In this rather small study, only 1 case of IH occurred [17].

The IH incidence in our cohort before routine closure is significantly lower than most rates reported in the literature. Although differences in follow-up could account for some discrepancies, this is unlikely due to our center’s solid protocols. Patients are included in the Dutch Audit for Treatment of Obesity (DATO), ensuring a high follow-up rate, particularly in the first three years. In 2021–2022, 98% remained in follow-up at 1 year, with 81–82% at 3 years, well above national averages. Additionally, bariatric surgery patients in the Netherlands are routinely referred back to their primary hospital for complications, ensuring continuity of care and accurate tracking of IH, which makes poor follow-up an unlikely cause for the low IH incidence. While potential explanations for our low rates, such as a more selective approach to reoperations or differences in terminology, could be considered, the fact remains that we cannot conclusively explain these low rates. However, the consistency in rates both before and after closure suggests that these findings are reliable.

While studies with low pre-closure IH rates report a significant reduction in IH after closure [18, 19], we observed no such decrease in our cohort. Although the lack of reduction might initially raise concerns about closure technique, Aghajani et al. demonstrated that endohernia stapling, the technique we used, effectively reduces IH compared to non-closure, while Muir et al. found no difference in IH rates between stapling and suturing [20]. The frequent reopening of MDs in our cohort—observed in 90% of reoperations—may better explain this lack of effect, suggesting that recurrent defects, rather than the closure technique, are responsible for the absence of IH reduction.

We observed kinking of the JJ in 0.84% of our patients. Although this rate is low, kinking results in prolonged hospital stays and increased ICU admissions, warranting careful consideration against the benefits of routine closure. Kinking is likely associated with the closure of the JJ defect, as its anatomical proximity to the anastomosis increases the risk of distortion or tension. In contrast, closure of Petersen’s defect is anatomically distant and unlikely to directly contribute to kinking. Hajibandeh et al. reported increased small bowel obstruction risk, including JJ kinking [21], and Stenberg et al. reported a significant increase in kinking within 30 days postoperatively [15]. While they argue that the lower long-term small bowel obstruction rate outweighs this, ICU admissions and costs were not addressed. Aghajani et al. proposed a straightforward intervention to mitigate kinking without introducing additional complications [8]. However, in our center, we refrained from further dividing the mesentery as described by them, citing concerns over potential risks such as bleeding or ischemia. Nonetheless, the efficacy of this approach warrants further investigation.

A positive CT scan for IH was the only significant predictor in multivariable analysis. Our previous study also identified CT as a significant predictor [7], and Nawas et al. confirmed the diagnostic accuracy of abdominal CT in a recent review [22]. Despite varying CT sensitivity reports in literature [15, 18, 19, 23, 24], we found that 74% of patients who underwent reoperation after a negative CT scan reported pain relief, although only 13% had perioperative IH. Postoperative pain relief in patients without perioperative IH might be attributed to several factors. Some patients may have had intermittent IH that was not confirmed on CT scan but was resolved by closing the MD during surgery. This illustrates the complexity of postoperative pain management and underscores the limitations of CT as a diagnostic tool.

## Limitations

This study has several limitations. The retrospective design introduces potential selection bias and reliance on available medical records. However, selection bias was minimized by systematically including all relevant cases from our surgical data in the medical records. Standardized follow-up is highly emphasized in our center, ensuring reliable data collection. The single-center analysis might limit the generalizability of the results, though the high volume of annually conducted surgeries helps mitigate this issue.

Variability in the definition and diagnosis of IH across different studies and clinical practices could impact the comparability of results. Nevertheless, this does not affect the comparison with our pre-closure results, as the same definitions were used consistently.

The limited follow-up duration in our cohort after closure may not capture long-term outcomes and complications associated with routine closure of mesenteric defects. This limitation could potentially result in a higher number of IH cases after routine closure, underscoring the lack of beneficial effects in our cohort.

Variability in surgical and closure techniques may impact the consistency and outcomes of the procedure. However, at our institution, RYGB and reoperations are conducted by three experienced bariatric surgeons who follow the same protocol, minimizing inter-surgeon variability. Analysis of our cohort revealed no statistically significant differences between surgeons.

## Conclusion

This retrospective cohort study in our high-volume bariatric surgery center indicates that routine closure of MD using endohernia staples (ETHICON, USA) during LRYGB does not reduce the incidence of IH but introduces the new complication of kinking of the JJ in 0.84% of patients, leading to prolonged hospital stays and increased ICU admissions. Postoperative pain relief rates were similar regardless of MD closure. Although CT scans predicted IH, they cannot replace the necessity of clinical expertise.

Our findings highlight the need for more high-level evidence on the benefits and potential risks of routinely closing of MD. This study emphasizes the complexities of optimizing surgical techniques to minimize IH risk while avoiding JJ complications. Future research should explore alternative closure methods, such as the division of the jejunal mesentery for a distance of 4–5 cm, and investigate strategies to enhance diagnostic accuracy beyond CT imaging alone.

## Data Availability

The research data supporting the results of this manuscript are available upon reasonable request from the corresponding author.
